# Multinucleate cell angiohistiocytoma: a diagnostic challenge^⋆^

**DOI:** 10.1016/j.abd.2022.01.017

**Published:** 2023-05-22

**Authors:** Patricia Mayumi Ogawa, Maria Cristina Arci Santos, Nilceo Schwery Michalany, Renato Shintani Hikawa

**Affiliations:** a Department of Dermatology, Universidade Federal de São Paulo, Escola Paulista de Medicina, São Paulo, SP, Brazil; b Department of Pathology, Universidade Federal de São Paulo, Escola Paulista de Medicina, São Paulo, SP, Brazil

Dear Editor,

A 33-year-old male patient presented with multiple erythematous-brown papules measuring 1 to 5 mm, disseminated on the upper limbs, trunk and thighs (Fig. 1). Dermoscopy disclosed the presence of a peripheral network and a central homogeneous pink area (Fig. 2). The condition had started two years before when he sought another Service and received a diagnosis of secondary syphilis, confirmed by non-treponemal (VDRL 1:64) and treponemal tests (positive FTA-ABS). He was treated on two occasions with three doses of benzathine penicillin, with no improvement of the skin condition (VDRL after treatments - 1:16). Diagnostic hypotheses were made of generalized multinucleate cell angiohistiocytoma (MCA) and eruptive dermatofibromas.

**Figure 1:**
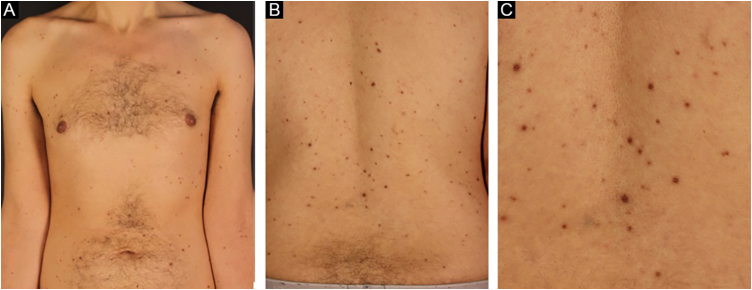
Brownish-erythematous papules affecting the upper limbs and trunk (A and B) and at closer view (C)

**Figure 2:**
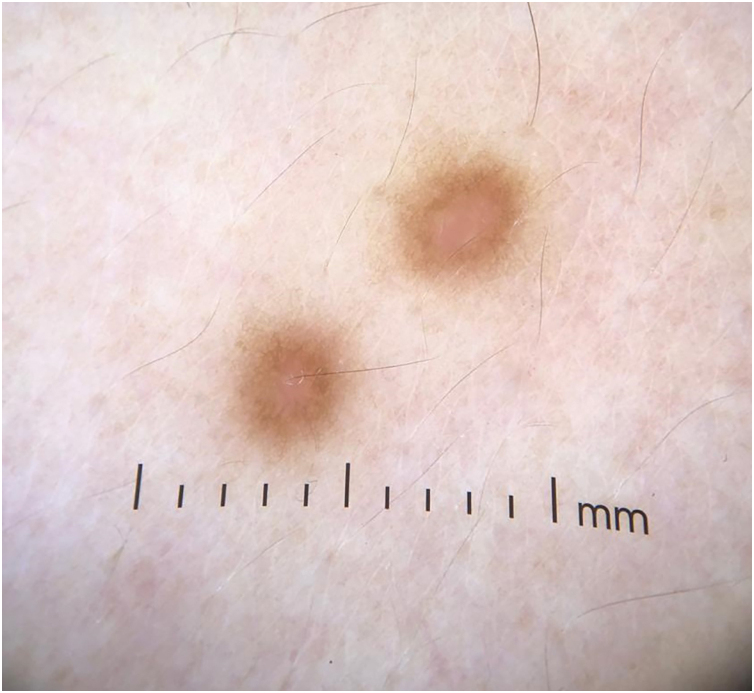
Dermoscopy showing a peripheral network with a central homogeneous pink area

Histopathology showed proliferated capillary vessels with dilated lumen (Fig. 3A) and multinucleate giant cells with ample cytoplasm interspersed with fibroblasts, lymphocytes, and histiocytes (Figs. 3B and 3C). Immunohistochemical analysis showed: immunoexpression of CD138 in the cytoplasmic membrane of the interstitial plasmocytes that permeated the lymphoid aggregates (Fig. 4A); cytoplasmic immunoexpression of Factor XIIIa in spiky multinucleate giant cells (Fig. 4B); positive expression of CD68 and CD163 in dermal histiocytes and negative in endothelial cells; CD31 positivity in the vascular component and negative immunoexpression of S100, establishing the diagnosis of MCA.

**Figure 3:**
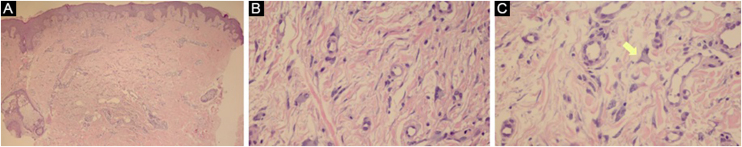
(A) The superficial reticular dermis shows proliferated capillaries with dilated lumen (Hematoxylin & eosin, ×40). (B) In some regions, multinucleate cells with ample cytoplasm interspersed with fibroblasts, lymphocytes, and histiocytes are observed. (Hematoxylin & eosin, ×400). (C) Some multinucleate cells have a spiky cytoplasm (arrow; Hematoxylin & eosin, ×400)

**Figure 4:**
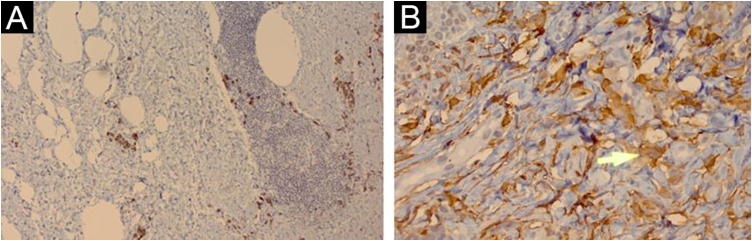
(A) Immunohistochemical analysis showing immunoexpression of CD138 in the cytoplasmic membrane of interstitial plasma cells that permeate the lymphoid aggregates (×100). (B) Cytoplasmic immunoexpression of factor XIIIa in spiky multinucleate cells (arrow; ×400)

After he was instructed about the condition, the patient chose not to undergo treatment for aesthetic purposes and returned after one year with spontaneous significant improvement in the number, pigmentation, and size of the lesions (Fig. 5).

**Figure 5:**
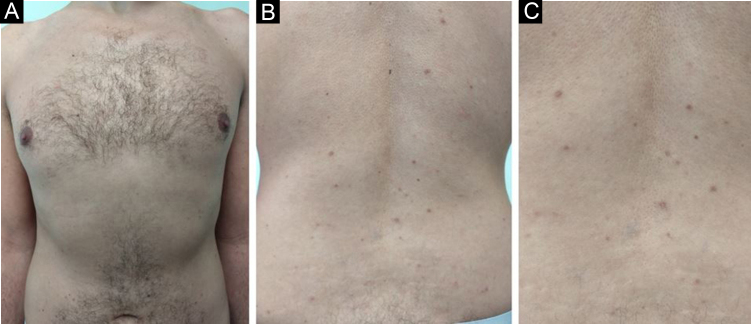
Decrease in the number, pigmentation and size of the lesions after one year in the anterior thoracic region (A) and back region (B and C).

## Discussion

Multinucleate cell angiohistiocytoma is a rare benign fibrous histiocytic proliferation, described in 1985.^1^ It often presents as well-circumscribed, dome-shaped erythematous-brown papules clustered on the hands, wrists, face, or legs. Localized, multifocal and generalized variants have been described, with the localized variant being the most common. Approximately 150 reported cases were found, with only 15 of the generalized variant.^2^ There is an important female predilection in the localized and multifocal variants, whereas males and females are equally affected in the generalized variant.^3^

Since the first description of MCA, several hypotheses have been proposed for its etiology and pathogenesis. Most authors believe it to be a reactive process and reports of spontaneous remission further support this hypothesis.^4^, ^5^ The reported case may represent MCA manifesting after *Treponema pallidum* infection. Another theory proposes that MCA arises under the influence of female hormones due to the identification of estrogen receptor alpha expression in the interstitial and multinucleate cells of lesions in several patients.^6^ This theory would also explain why MCA occurs more frequently in women in the localized and multifocal variants. Other authors consider MCA a variant of dermatofibroma^7^ or associate it with an altered immune status.^2^

Dermoscopy can show some similarities with dermatofibromas, such as whitish areas and a fine peripheral reticulated pattern. Diffuse reddish areas with blurred edges, which likely represent the characteristic vascular dilation, may also be found.^8^

Histopathology shows proliferation of dilated capillaries, fibrous stroma with thickened collagen bundles, and the presence of multinucleate giant cells. The overlying epidermis may be normal or hyperplastic. The presence of at least three of the described in the adequate clinical context is highly specific for MCA and allows differentiation from fibrous papule and dermatofibroma. The criteria considered for the diagnosis were: a) Presence of atypical and multinucleate fibroblasts with at least two nuclei; b) Presence of superficial parallel dermal fibrosis; c) Presence of thickened superficial papillary dermal vessels; d) Absence of perifollicular fibrosis.^9^

Regarding immunohistochemistry, the multinucleate cells are typically negative for factor VIII and CD34 and positive for vimentin and factor XIIIa. There is a positive correlation between CD68-positive expression of endothelial cells and lesion size, and an inverse correlation between CD68-positive expression on multinucleate cells and the development of multiple lesions.^4^

The biological behavior of MCA is progressive in most cases, albeit benign, with spontaneous improvement being rare. Treatment can be carried out for aesthetic purposes. The currently available therapeutic options include topical or intralesional corticosteroids, surgical excision, cryotherapy, argon laser, intense pulsed light, and CO_2_ laser.^10^

In conclusion, the authors describe a rare case of MCA possibly triggered by *Treponema pallidum* infection and suspected by dermoscopic findings. Because it is rare, it is important that dermatologists and pathologists are able to recognize it for a better approach and to provide recommendations to the patient. Multiple treatment options are available to improve the aesthetic appearance, most of which are supported by case reports.

## Financial support

None declared.

## Authors' contributions

Patricia Mayumi Ogawa: Design and planning of the study; drafting and editing of the manuscript; collection, analysis and interpretation of data; critical review of the literature.

Maria Cristina Arci Santos: Approval of the final version of the manuscript; drafting and editing of the manuscript; collection, analysis and interpretation of data; effective participation in research orientation; critical review of the manuscript.

Nilceo Schwery Michalany: Approval of the final version of the manuscript; collection, analysis and interpretation of data; intellectual participation in the propaedeutic and/or therapeutic conduct of the studied cases; effective participation in research orientation; critical review of the manuscript.

Renato Shintani Hikawa: Approval of the final version of the manuscript; collection, analysis and interpretation of data; design and planning of the study; intellectual participation in propaedeutic and/or therapeutic conduct of studied cases; effective participation in research orientation; critical review of the manuscript.

## Conflicts of interest

None declared.
